# The Breathing IQ: an anthropometric index of diaphragmatic breathing efficiency

**DOI:** 10.3389/fphys.2024.1394109

**Published:** 2025-04-02

**Authors:** Belisa Vranich, Rachel Lee, John Zapanta, Brian Shoen, Helene Houghton, Crystal Ruelas, Mary Lisa Wahlfeldt, Robin Kerr, Andrei Pripotnev, Michaela Goorahoo, Jean-Berne Baptiste, Joanne Elliot

**Affiliations:** The Breathing Class, San Diego, CA, United States

**Keywords:** breathing pattern dysfunction, diaphragmatic breathing, breathing exercises, biomechanics, respiratory health

## Abstract

**Background:**

Breathing pattern disorders (BPDs) are becoming increasingly relevant due to the rise of respiratory illnesses but are often limited to diagnoses of hyperventilation syndrome and do not consider breathing dysfunction of mechanical origin. Impaired diaphragmatic and inefficient breathing patterns often evolve into downstream musculoskeletal and psychological consequences. Respiratory research has consistently called for the standardization of methods that can consider breathing as multifactorial and also consider breathing mechanics.

**Aim:**

This paper aims to introduce the Breathing IQ (BIQ) as a novel anthropometric index of abdominothoracic flexibility for identifying biomechanical breathing patterns and assessing diaphragmatic breathing efficiency.

**Method:**

A sample of N = 384 individuals was assessed with the BIQ pre- and post-intervention of five corrective exercises in a single 90-minute session to identify changes in the BIQ grade (A–F), as determined by measuring the breathing range of motion (ROM) and location of movement (LOM).

**Results:**

Binary-grade improvement (yes/no) occurred in 331 of 370 without an A grade at baseline (89.5%), p < 0.001. Before the intervention, 249 (64.8%) were graded an F and only 14 (3.7%) were graded an A. After the intervention, only 20 (5.2%) were graded an F and 102 (26.6%) were graded an A. Breath hold (BH) improved from pre- to post-intervention, from 37.2 (18.7) to 66.0 (26.9), with a mean change 24.3 (15.3), which was highly significant (p < 0.001).

**Conclusion:**

The BIQ shows preliminary potential as an effective screening tool for mechanical breathing dysfunction.

## Introduction

Attention to breathing pattern disorders (BPD) has grown in the last decade due to ongoing respiratory health crises caused by viruses, the rise of COPD, airborne environmental toxins, and increasing attention to stress and mental health. BPD is generally defined as “a group of breathing disorders in patients where chronic changes in breathing pattern result in dyspnea and often non-respiratory symptoms in the absence of, or in excess of, organic respiratory disease” (Boulding et al., 2016). Although subtypes of BPD include hyperventilation syndrome, periodic deep sighing, thoracic dominant breathing, forced abdominal expiration linked to COPD, and thoraco-abdominal asynchrony (Boulding et al., 2016), the term BPD has traditionally been used interchangeably with “hyperventilation” and “over-breathing,” which affects 9% of the general adult population (Jones et al., 2013). Individuals diagnosed with hyperventilation syndrome are usually female, in their 20s, with co-occurring psychiatric anxiety (Pfortmueller et al., 2015). However, research suggests that a more comprehensive, multifactorial definition is in order, which includes biomechanical issues (Courtney, 2017).

Breathlessness, dyspnea, or “air hunger” is the most common symptom reported by patients. Although its origin eludes us, experts suggest that “it stems from biomechanical or psychological disturbances, or some combination of the two. One possible reason is breathing that stems from the upper chest rather than the entire chest and abdomen … Treatment for dysfunctional breathing is not standardized yet” (Lloyd, 2017, p. 27). This idea has been reiterated throughout respiratory health research (Courtney et al., 2011; De Peuter et al., 2004; American Thoracic Society, 1998).

Due to BPD’s traditionally limited definition, the rate of BPD that includes other subtypes may be higher in the general population. Alarming but still not well-recognized is the high incidence of biomechanical problems in breathing. A recent study in Japan found that of 1933 student athletes, less than 10% were diaphragmatic breathers (Shimozawa et al., 2023). In the general population, cumulative respiratory stressors have decreased abdominal and diaphragmatic responsibility, which leads to more apical breathing. For example, a sustained posture of “bracing” results in reduced chest wall mobility, which results in the diaphragm “shortening” (Cassart, et al., 1997; Laghi and Tobin, 2003), becoming “inhibited,” “amnesic,” even “paralyzed.” This, in turn, affects the tidal volume (Wade, 1954) and can initiate a vicious circle of shallow, fast, auxiliary muscle-driven breath.

These impaired diaphragmatic and suboptimal breathing patterns often evolve into downstream musculoskeletal and psychological consequences such as chronic back and neck pain and injury (Clifton-Smith, 2014; Hodges et al., 2001), high blood pressure (Radaelli et al., 2023), acid reflux (Eherer et al., 2012), reduced HRV (Courtney et al., 2011), and panic attacks (Fried, 1999), whose origins are often not attributed to the source—the breath. Progress is being made in the field of psychology, where there is a significant amount of research focusing on breathing in the treatment of stress, anxiety, and depression (Gerbarg et al., 2019; Fried, 1999; Jerath et al., 2015; Fonkoue et al., 2020; Sarno, 1999). The diaphragm’s relationship with the vagus nerve is important for regulating communication with the parasympathetic nervous system (Bordoni et al., 2018).

The most commonly used breathing screening in clinical and emergency settings is the rate of breath, despite having significant potential for errors (Drummond et al., 2020). Studies have found that the assessment of breath movement by simple observation is poor (Spiteri et al., 1988; Perkins et al., 2005). Evaluating only the rate of breath does not consider the mechanical impacts of breathing, despite their direct impacts on the biochemistry and psychology (Courtney, 2017).

Experts in the fields of posture, yoga, Pilates, dance, and movement methods such as Feldenkrais have been addressing the importance of breathing mechanics for decades (Bowman, 2014; Farhi, 1996; Calais-Germain, 2006; Miller, 2023; Makovsky, 2023). The inclusion of mechanical correctives focusing on the “location” of the breath and chest mobility in breathing science (Hendricks, 2005; Dooley, 2014) is part of the breath retraining of the BradCliff Method (Bradley, 2002), the Postural Restoration Institute (Boyle et al., 2010), dynamic neuromuscular stabilization (Mahdieh et al., 2020), and the Egoscue Method (Fifer et al., 2017). Other researchers have considered the assessment of muscle dominance (Peper, et al., 2016; Wade, 1954), agreeing that thoracic excursion is an important tool in assessing dysfunctional respiratory patterns (Reddy et al., 2019).

Optimal breathing involves the efficient use of the diaphragm, the primary breathing muscle, and has been linked to a multitude of physical and mental benefits that range from improved cerebral spinal fluid movement (Bordoni et al., 2018; Dreha-Kulaczewski et al., 2015), reduced exercise-induced oxidative stress (Martarelli et al., 2011), improved sleep quality (Liu et al., 2020), reduced digestive disorders such as acid reflux and GERD (Eherer et al., 2012), lowering the perception of pain (Busch et al., 2012; Peper et al., 2006), and good postural control (Boyle et al., 2010; Kolár et al., 2012; Koseki et al., 2019).

However, there is a need for an accessible, cost-effective, noninvasive tool to measure the diaphragm’s efficiency in breathing. Currently, ultrasound is the most commonly used method for evaluating diaphragm movement (Technical report, 2021; Houston et al., 1994; Wait et al., 1989; Cohen et al., 1994; Le Neindre et al., 2016). Other techniques for evaluating the diaphragm, such as the sniff test for chest fluoroscopy, have drawbacks that include operator dependence and cost. The development of the Manual Assessment of Respiratory Motion (MARM) was inspired by the need for less invasive techniques to evaluate breathing mechanics and was even found to be superior to respiratory inductive plethysmography (Courtney et al., 2008). Abnormal scores on the MARM are correlated with apical breathing patterns, disproportionate dyspnea, and other medically unexplained symptoms (Courtney et al., 2011b).

Thoracic excursion is a measurement of chest expansion during inhalation and exhalation to estimate respiratory function. It was first noted in the academic literature in the late 1800s to describe the progression of tuberculosis patients and has since grown to be used globally for the evaluation of respiratory ailments (Adedoyin et al., 2010; Illeez et al., 2016) and as a measure of disease and treatment response in patients with scoliosis (Burgos-Vargas et al., 1993), cystic fibrosis, and ankylosing spondylitis (Custers et al., 2005; Nishigaki et al., 2013). Thoracic excursion provides valuable clinical insights into lung function, specifically vital capacity (Debouche et al., 2016; Reddy, et al., 2019), tidal volume (Padasdao et al., 2013), and respiratory muscle strength (Cordoba Lanza et al., 2013). Tidal volume has been found to have a linear relationship with ultrasound measurements of diaphragm excursion (Cohen et al., 1994; El-Halaby et al., 2016).

Although thoracic excursion is reliable and has good interrater reliability (Reddy, et al., 2019), the methodology is vastly inconsistent. The location of measurement has ranged from the axilla to 10th rib, with some studies measuring patients while standing and others while supine. Not only does the position of the measuring tape and the posture of the patient need to be standardized (Harris et al., 1997) but also the varying instruction verbiage has been observed to significantly affect the outcome (Fagevik Olsen, 2011).

Additionally, research lacks consensus in determining a range of normal thoracic excursion measurement results in the general population to compare treatment efficacy. Reference range values vary from 2 cm to 8.48 cm (Carlson, 1973; Bockenhauer et al., 2007; Adedoyin and Adeleke, 2012; Finsback and Mannerkorpi, 2005; Chilton-Mitchell et al., 2013; Cidem et al., 2012; LaPier, 2002). Well-conditioned individuals may have higher numbers, e.g., “singers and mountaineers can go up to 6 inches” (Prudden, 2016).

Instead of using an absolute range, the Breathing IQ (BIQ) uses a percentage to reflect the difference between inhalation and exhalation, as demonstrated by physical therapists (Paula Moore, 2018; Dr. Rene Cailliet 1987). Using a percentage easily solves the dilemma of controlling for varying body types, ensuring that the focus is on the patients’ own baseline and progress is gauged from there.

The BIQ, a novel anthropometric index of diaphragmatic breathing efficiency, addresses this problem by combining well-established thoracic excursion measurements with the location of movement of the breath. The BIQ also standardizes the location of the measurement. The measurement of the expansion of the lower chest wall (Reddy et al., 2019) at the abdominal level (Malaguti et al., 2009) has found a better correlation with lung function than the historically standard axillary and xiphisternal measurements. Research supports measuring thoracic excursion specifically at the 10th rib and only one plane of measurement (Moll and Wright, 1972). Standardizing the location of measurement to this easy anatomical landmark allows the measurement to be done by any gender examiner and in a public setting; it also enables patients to continue measuring their progress because of the ease and intuitive nature of this body landmark.

In addition to quantifying thoracic excursion, the BIQ assesses the muscles responsible for initiating the breath, or location of movement, as emphasized in previous studies (Peper, et al., 2006; Wade, 1954; Chapman et al., 2016; Miller, 2023). The literature suggests the need for an updated system of classification, which also includes other disturbances, such as thoracic dominant breathing (Boulding et al., 2016). Even with adequate thoracic excursion, reliance on accessory breathing muscles like the trapezius, sternocleidomastoid (SCM), serratus anterior, pectoralis minor, and scalenes can hinder efficient diaphragmatic breathing and make accurate measurements of diaphragmatic-driven thoracic excursion challenging (Wade, 1954). The BIQ evaluates diaphragmatic breathing efficiency by analyzing both thoracic excursion and the dominant muscles used for breathing. It grades individuals by cross-referencing the location of movement (LOM) and range of motion (ROM) of their breath, assigning a score from A to F).

The BIQ differentiates between apical breathing and efficient diaphragm-driven breathing. Efficient diaphragmatic breathing has both anterior abdominal wall and circumferential lower rib cage movement due to contributions from the crural and costal diaphragm and the lower intercostals. During inhalation, the diaphragm moves caudally toward the pelvic floor, flattens, and gently compresses the underlying organs (Liu et al., 2024). The lower ribcage should move proportionately and symmetrically in a lateral, ventral, and dorsal direction, expanding the space between the intercostals, while the abdominal walls should expand in a cylindrical fashion (Reyes et al., 2017; Perri and Halford, 2004). This breath is more efficient due to its larger volume and lower energy requirements, allows for appropriate metabolically contextual flexibility, encourages a more balanced inhale and exhale, promoting greater biochemical equilibrium, is responsive in managing arousal, and can be slowed to the rate of coherence or resonance. When trained, efficient diaphragmatic breathing can delay fatigue and significantly enhance athletic performance (McConnell, 2011).

The BIQ helps standardize the assessment of efficient diaphragmatic breathing. Directives to “breathe diaphragmatically,” “take a deep breath,” or perform “deep slow breathing” are included in many clinical settings, including COPD respiratory rehab, relaxation therapy, and even cancer radiation treatment protocols. Patients may be asked to “take a belly breath” or have their “abdomen expand” in an attempt to lower the rate of breath (Ma et al., 2017). The terms “belly,” “diaphragmatic,” or “abdominal breathing” (Hagman et al., 2011; Perri and Halford, 2004; Liebenson, 2007; Clifton-Smith, 2014; Roussel et al., 2009) have been used interchangeably, although belly expansion without rib cage movement could point to a chest wall dysfunction (Chapman et al., 2016). Unfortunately, these directives cause a large amount of variance in the resulting breath (Moll and Wright, 1972).

The BIQ also includes correctives to restore optimally efficient diaphragmatic breathing, achieved by an “A” grade. Five exercises are repeated with the goal of optimizing abdominothoracic respiratory flexibility. Stretching of respiratory muscles improves lung capacity (Putt et al., 2008) and their length, mobility, and power, which further improves ventilation and oxygenation (Yokoyama et al., 2022). These exercises address the pitfalls in standard pulmonary rehabilitation programs consisting of aerobic physical conditioning exercises, despite other benefits, which have not been shown to modify lung function (Putt et al., 2008; Rehman et al., 2020). Incentive spirometers and inspiratory muscle training devices are the most commonly used practices post-surgery, yet neither includes standardized instructions specifically encouraging measurable diaphragmatic breathing (Franklin and Anjum, 2023).

This preliminary study has two aims: first, to introduce the Breathing IQ as a modern anthropometric index of abdominothoracic flexibility and diaphragmatic breathing efficiency, which offers information on the biomechanical aspect of BPD. The importance of dysfunctional breathing biomechanics is drawing increased attention as recent findings have demonstrated that even with a normal respiratory rate, the most common respiratory assessment, individuals can have high-risk mechanical patterns that correlate with the severity of illness (Ashe et al., 2024). Because the diaphragm plays multiple roles in the body, including detoxification, balance, and nervous system regulation, a functional, noninvasive, and cost-effective assessment providing immediately actionable correctives and progress tracking could provide invaluable health information. Second, we respond to suggestions from previous thoracic excursion studies (Carlson, 1973; Bockenhauer et al., 2007), pointing out the need for a gold standard for the assessment of biomechanical dysfunction in breathing disorders. Prior research highlights the lack of standardization in instructions, including verbal cues, measurement location and position, and directives, making it difficult to track efficacy across treatments and baselines for different population subgroups.

## Methodology

Ten examiners were trained and certified in the BIQ methodology and treated a total of 384 participants (see Table 1 for participant data). Each examiner individually treated 20 subjects. Participants qualified for the study if they were at least 18 years old at the time of assessment, reported no breathing difficulties, and were not currently under the care of a medical professional for any respiratory problem. Exclusion criteria included a history of musculoskeletal or any condition that would prevent them from standing for the thoracic excursion measurement. For each subject, examiners administered a health intake questionnaire, measured pre- and post-scores of the BIQ and breath hold (BH) time in seconds, and conducted an intervention of five corrective exercises in a 90-min session. The training program for BIQ certification is recognized by the Global Professional Breathwork Alliance and International Breathwork Foundation.

**TABLE 1:** Participant characteristics, Range of Motion (ROM), and Breathing Hold (BH) changes by sex.

Variable	^a^Both sexes combined (N = 384)	Male (N = 121)	Female (N = 262)
Age, mean (SD)	44.2 (17.0)	40.9 (15.0)	45.8 (17.7)
Age group			
<30	82 (22.2)	29 (24.8)	53 (21.0)
30–39	67 (18.1)	28 (23.9)	38 (15.1)
40–49	78 (21.1)	27 (23.1)	51 (20.2)
50–59	71 (19.2)	20 (17.1)	51 (20.2)
60+	72 (19.5)	13 (11.1)	59 (23.4)
ROM (pre)	44.2 (36.5)	48.8 (39.6)	42.0 (34.9)
ROM (post)	86.5 (32.9)	87.2 (35.9)	86.1 (31.6)
ROM change, mean (SD); p-value	42.3 (26.9); <0.001	38.4 (28.5); <0.001	44.1 (26.1); <0.001
BH (pre)	37.2 (18.7)	40.3 (21.0)	34.7 (16.7)
BH (post)	66.0 (26.9)	76.1 (28.9)	58.2 (22.6)
BH change, mean (SD); p-value	24.3 (15.3); <0.001	29.9 (13.4); <0.001	20.0 (15.6); <0.001
^b^ Grade improved, n (%); p-value	331/370 (89.5%); <0.001	99/111 (89.2%); <0.001	232/258 (89.9%); <0.001

Participant data including age, range of motion (ROM), breathing hold (BH), and grade improvement by sex. Results are presented as means (SD) or percentages (%), with p-values for ROM and BH changes and grade improvement.

^*a*^ One subject’s sex was not reported.

^*b*^ 14 subjects with grade A at baseline were excluded.

**FIGURE 1:**
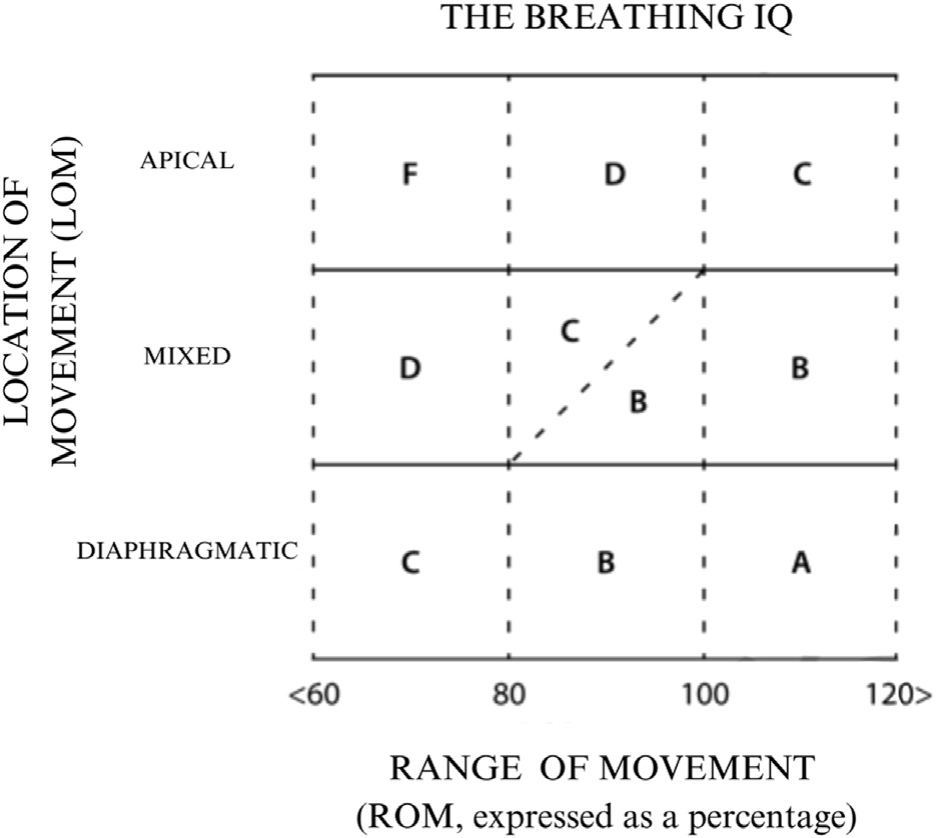
Breathing IQ (BIQ) grading diagram. The grade is determined by the intersection of the participant’s LOM and ROM scores.

The BIQ is calculated by combining the LOM and ROM, which results in a grade ranging from A to an F (Figure 1).

**FIGURE 2:**
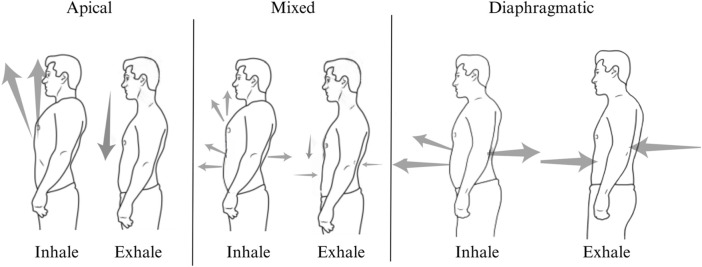
LOM styles include apical, mixed, and diaphragmatic movement.

**FIGURE 3:**
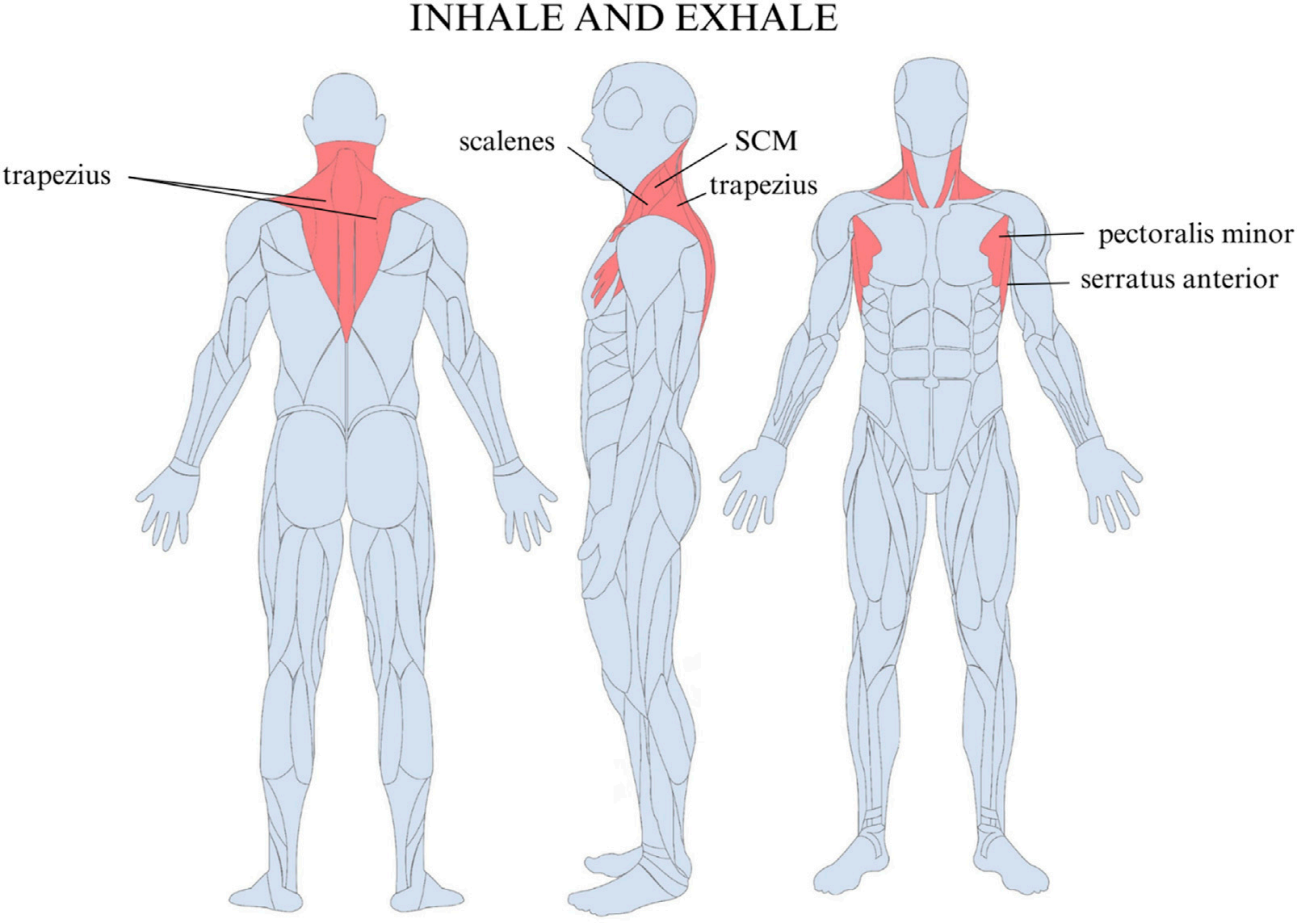
Muscles used in apical breathing include the trapezius, sternocleidomastoid (SCM), serratus anterior, pectoralis minor, and scalene muscles.

**FIGURE 4:**
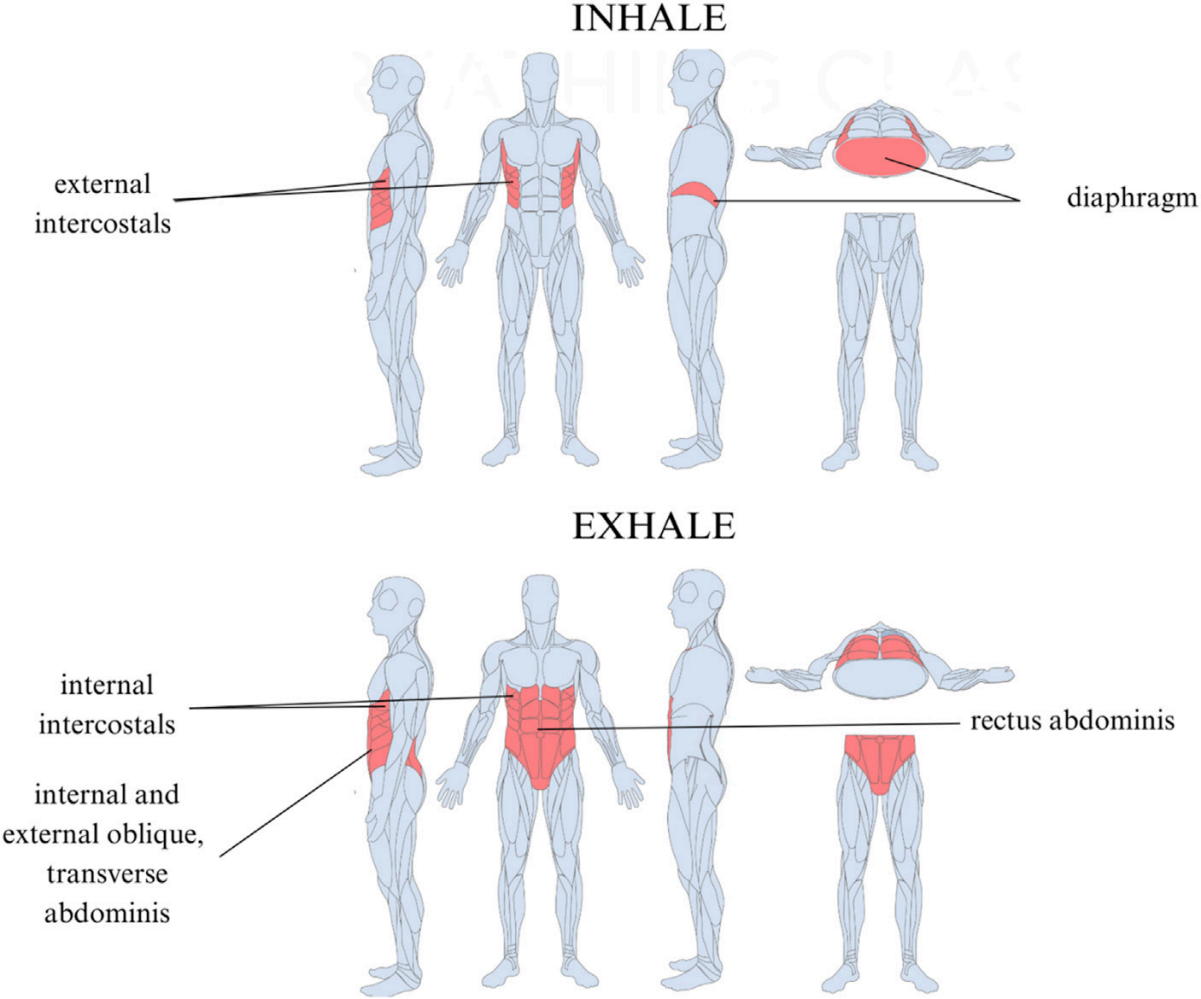
Muscles used in efficient diaphragmatic breathing. Inhalation is driven by the diaphragm and external intercostals. Exhalation is controlled by the internal intercostals, internal and external oblique, transverse abdominis, and rectus abdominis.

The LOM was ascertained by delivering scripted instructions and visually observing the activation and sequencing of breathing musculature during three maximal inhalations and exhalations in standing participants. There are three possible LOMs, namely, apical, diaphragmatic, or mixed (Figure 2). An apical breath is identified when there is minimal to no expansion of the abdomen and lower ribcage, and auxiliary muscles dominate the initiation and movement of the breath, which can be identified with the vertical movement of the clavicles, the upper rib cage superior to the level of the xiphoid process, and shoulders (Figure 3). Diaphragmatic LOM is marked by breathing initiated by the anterior, lateral, and posterior movement of the abdomen and lower thoracic cavities and an absence of apical movement (Figure 4). A mixed LOM is determined when aspects of both breathing styles are utilized, which usually presents as noticeable apical auxiliary muscle movement accompanied by anterior abdominal expansion, but with a lack of lateral low ribcage movement.

**FIGURE 5:**
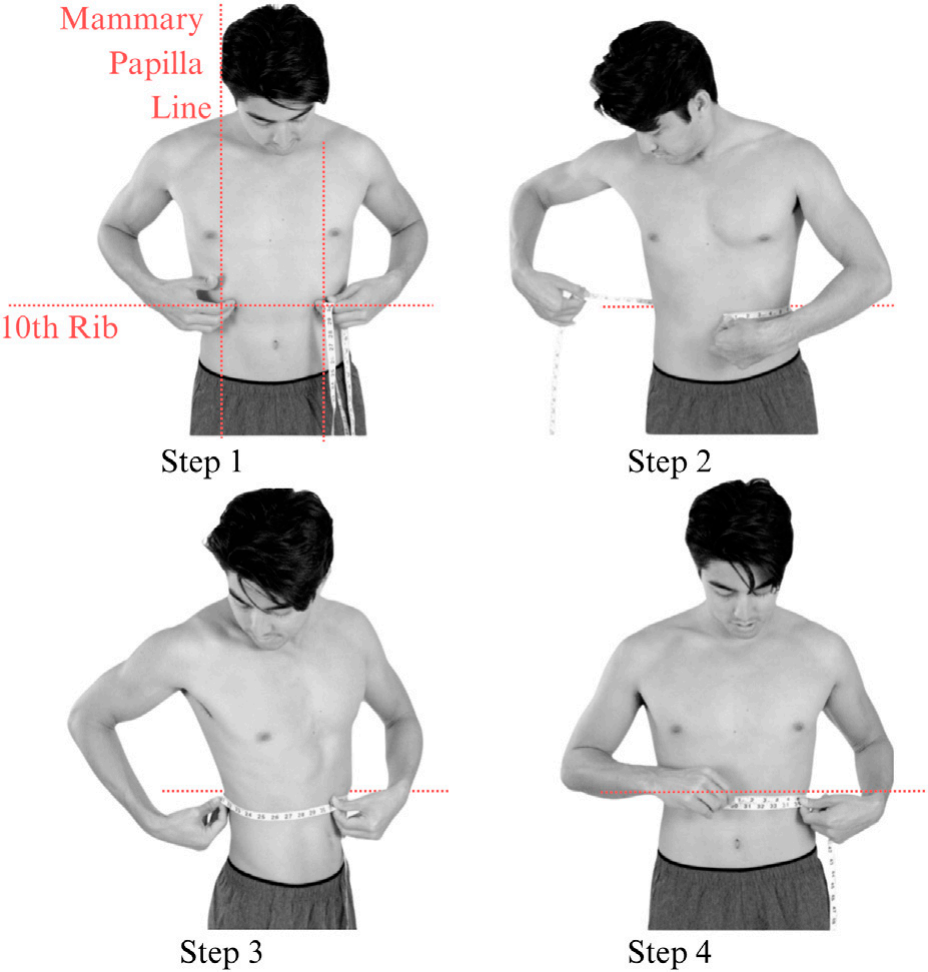
Placement of tape measure while examining the ROM. The tape measurement can be taken on top of non-bulky clothing.

ROM was then evaluated in a standing position in all participants by placing a soft measurement tape on the crossing point between the vertical mammary papilla line and the lower end of the rib cage and wrapping it around the subject to measure the circumference of three maximal inhalations and exhalations and recording the mean (Figure 5). Participants’ measurements were taken on the top of form-fitting clothing.

The ROM was calculated using the below equations based on thoracic excursion measurements and standardized to result in a percentage:

$$\begin{array}{c} \text{Inhale }‐\text{ Exhale}=\text{Difference}\\ \frac{\text{Difference}}{\text{Exhale}}\;X\;1000=\text{ROM} \end{array}$$

The corrective exercise intervention was conducted within 10 minutes after the pre-assessments and included five movements to optimize abdominothoracic respiratory flexibility (Table 1). Stretching of respiratory muscles improves forced vital capacity (Putt et al., 2008) and their length, mobility, and power, which further enhances ventilation and oxygenation. The five movements and stretches are “diaphragm extensions,” “rock and roll,” “intercostal stretch,” “spinal twists,” and “cat-cow” (Table 2). A diaphragmatic breath is cued in each position from easiest (supine) to most difficult (standing). These stretches have been carefully chosen as part of a succession of movements that build upon success and target each of the problems that have led to compromised respiratory flexibility.

**TABLE 2:** The five corrective exercises taught during the intervention are, in part, based on research by Kakizaki et al. (1999).

Exercise number	Exercise name	Description	Visual
1	Rock and roll	This exercise pairs inhalation and exhalation with an anterior and posterior pelvic tilt while seated, aiding in lowering the movement of the breath to encourage diaphragmatic awareness. It also connects the pelvic floor and the diaphragm	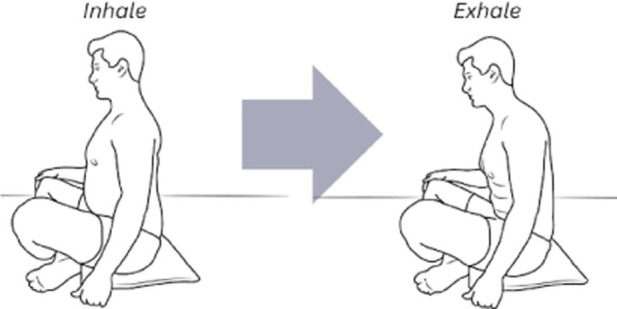
2	Diaphragm extensions	This exercise directs participants in a supine position to use the abdomen to push a weighted object upward during inhalation and allow it to descend during exhalation. Although the movements of the inhalation and exhalation remain the same as in the rock and roll, this supine position reduces unintentional initiation of the breath with the neck and shoulders	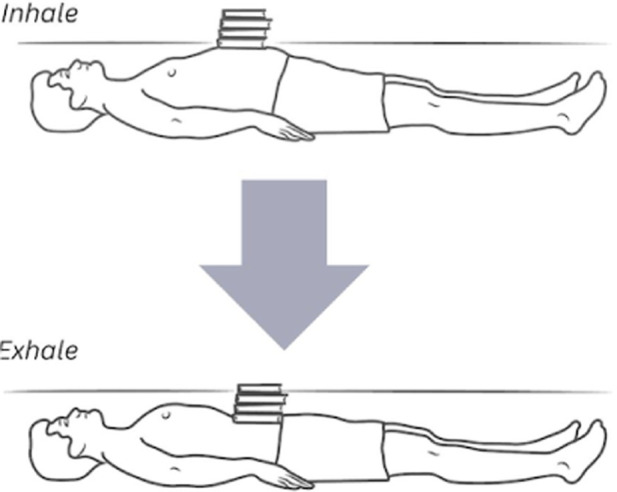
3	Cat-cow	This exercise is practiced on all fours. Exhalation is paired with a rounded back, dropped head, and active abdominal contraction. Inhalation is the opposite; the back is arched, the neck is craned, and the abdominothoracic cavity is relaxed. This exercise allows the patient to experience the breath as a full body movement with focus on hip rotation and efficient exhalation	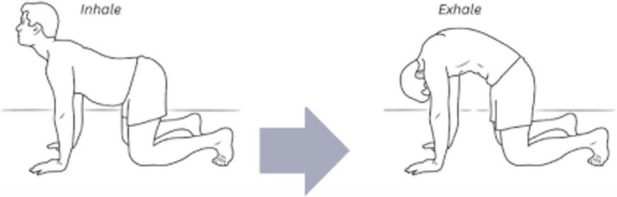
4	Intercostal stretch	This exercise encourages the opening of the ribcage by stretching the intercostals on an inhalation, seated, with forearm overhead. This exercise is pivotal in stretching tight obliques and intercostals, which are main causes of ribcage rigidity	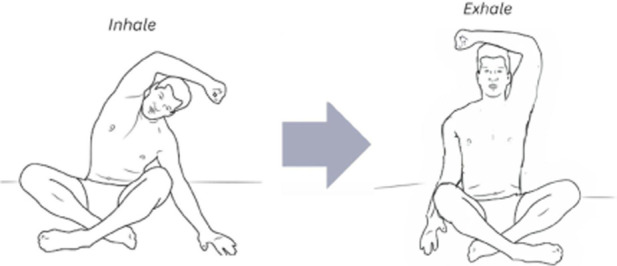
5	Thoracic twist	In a seated position, participants bend their knees, while the torso stretches 180° in the opposite direction. This more advanced exercise serves in both stretching the thoracic cavity and encouraging the individual to consciously initiate an efficient diaphragmatic breath despite being in a compressed position	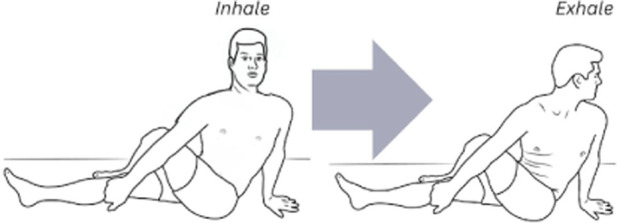

BH time was measured by determining the maximum number of seconds the participant could hold their breath after an inhale. The mean of three measurements was recorded. In studying hyperventilation syndrome and end-tidal CO_2_, Courtney et al. (2008) found that apical breathing patterns of individuals with abnormal spirometry correlated with lower breath hold times. Breath hold was included in this study to determine whether our results were in agreement with these findings.

## Statistical methods

Each subject was assessed before and after the intervention for LOM (apical, diaphragmatic, and mixed), ROM (expressed as a percentage), breath hold time in seconds, and BIQ grade (A–F) derived from the LOM and ROM. Descriptive statistics were produced on the aforementioned variables in addition to age. Cross-tabulations of pre- vs. post-intervention grade and LOM were computed. Paradoxical breathers (those with a negative ROM at baseline) were compared with non-paradoxical breathers on baseline grade and LOM via the chi-squared test. Changes in ROM and BH pre- vs. post-intervention were tested against 0 via the paired *t*-test. Grade improvement (binary) was tested against 0 via the Wilcoxon signed-rank test. Pearson’s correlation between age and ROM was computed and tested against 0 via Fisher’s z transformation.

In addition to the aforementioned analyses, the relationship between BIQ and age group was evaluated in frequency tables with chi-squared tests using pre-intervention data first and then post-intervention data. The relationship between BH and BIQ was assessed via ANOVA with a Bonferroni adjustment for multiple comparisons, using pre-intervention data first and then post-intervention data. The association between BIQ improvement (yes/no) and BH improvement (yes/no defined as an increase in BH) was explored via 2 by 2 tables using Fisher’s exact test. In BIQ improvement analyses, subjects with an A-grade pre-intervention were dropped as it was impossible for them to improve on BIQ. The above-stated analyses were performed in both sexes combined and then repeated in male and female participants separately. All analyses were performed using SAS v9.4 (SAS Institute, Cary, North Carolina).

## Results

Pre- and post-intervention breathing grades were assigned to subjects from 11 breathing instructors on a total of N = 384 subjects (121 male subjects, 262 female subjects, and 1 sex unstated). The results are summarized in Table 1. The mean age was 44.2 (SD 17.0), and age was evenly distributed amongst the five age groups. BH improved pre-to post-intervention, from 37.2 (18.7) to 66.0 (26.9), with a mean change of 24.3 (15.3), which was highly significant (p < 0.001). Binary-grade improvement (yes/no) occurred in 331 of the 370 without A grades at baseline (89.5%), p < 0.001.

The details of the grades from pre- to post-intervention are shown in Table 3. Before the intervention, 249 (64.8%) were graded an F and only 14 (3.7%) were graded an A. After the intervention, only 20 (5.2%) were graded an F, and 102 (26.6%) were graded an A. Only 1 subject showed a decline (moved from an A to a B after the intervention), and the vast majority improved (331 of the 370 without A grades at baseline improved [89.5%]).

**TABLE 3:** BIQ Grade Distribution Pre- and Post-Intervention.

Grade		Post					
		A	B	C	D	F	Total (col %)
Pre	A	13	1	0	0	0	14 (3.7%)
	B	19	3	0	0	0	22 (5.7%)
	C	10	18	7	0	0	35 (9.1%)
	D	21	12	22	9	0	64 (16.7%)
	F	39	61	68	61	20	249 (64.8%)
	Total (row %)	102 (26.6%)	95 (24.7%)	97 (25.3%)	70 (18.2%)	20 (5.2%)	384 (100.0%)

Distribution of BIQ grades before (Pre) and after (Post) the intervention, with row and column percentages displayed. Grades are categorized as A, B, C, D, and F, with total values provided for each column and row.

Table 4 shows a similar pattern of improvement occurred in the LOM. Of the 368 participants for whom LOM data were available, before the intervention, 250 (67.9%) were judged to be apical breathers and only 36 (9.8%) were judged to be diaphragmatic breathers (the remaining 82 were deemed mixed-type breathers). After the intervention, only 10 (2.7%) were judged apical breathers and 223 (60.6%) were judged diaphragmatic breathers (the remaining 135 were deemed mixed-type breathers). Only 2 subjects showed a decline (moved from diaphragmatic to mixed type after the intervention), and the vast majority improved (298 of the 332 non-diaphragmatic breathers at baseline improved at follow-up [89.8%]).

**TABLE 4:** LOM Distribution Pre‐ and Post‐Intervention by Breathing Pattern.

LOM pre/post	Diaphragmatic	Mixed	Apical	Total (col %)
Diaphragmatic	34	2	0	36 (9.8%)
Mixed	58	24	0	82 (22.3%)
Apical	131	109	10	250 (67.9%)
Total (row %)	223 (60.6%)	135 (36.7%)	10 (2.7%)	368 (100.0%)

^a^ 16 subjects had unreported LOM at baseline or post-intervention.

We found no association between BIQ and age group (p = 0.112 pre-intervention and 0.177 post-intervention). There was an association between BH and BIQ via ANOVA (Bonferroni-adjusted p-values <0.05 for A vs. C and A vs. F, with A showing longer BH) using pre-intervention data. Post-intervention, there were no F grades that also had BH data. Using grades A–D, there were no significant associations between BH and BIQ via ANOVA after Bonferroni adjustment; however, it might be noted that A vs. B and A vs. D were borderline significant (p < 0.1).

Finally, in sex-stratified analyses, the results of the abovementioned analyses were similar to the combined sex analysis, i.e., no substantial difference by sex.

## Discussion

The results of this preliminary study demonstrated that the BIQ could effectively measure changes in breathing mechanics in a single 90-minute intervention of corrective exercises, with the resulting breathing pattern being significantly more diaphragmatically driven. Before the intervention, 249 (64.8%) were graded an F and only 14 (3.7%) were graded an A. After the intervention, only 20 (5.2%) were graded an F and 102 (26.6%) were graded an A. Over the course of the intervention, 331 of 370 (89.5%) subjects without A grades at baseline improved.

The changes in the overall BIQ score were driven by significant improvements in LOM and ROM. Before the intervention, 250 (67.9%) participants demonstrated an apical breathing style and only 36 (9.8%) were judged to be diaphragmatic breathers, with the remaining 82 deemed mixed-type breathers. After the intervention, only 10 (2.7%) subjects were identified as apical breathers and 223 (60.6%) were judged to be diaphragmatic breathers, with the remaining 135 deemed mixed-type breathers. This highlights the efficacy of the corrective exercises in teaching participants to initiate breathing with the primary rather than auxiliary breathing muscles.

Prior to the intervention, the mean ROM was 44.2% (36.5). Post-intervention, the mean ROM increased to 86.5% (32.9) with a change of 42.3 (26.9), which was statistically significant (p < 0.001). The ROM is a reflection of the difference in abdominothoracic flexibility between inhalation and exhalation. The significant increase in the ROM can indicate an increased diaphragm and external intercostal expansion during the inhalation, a greater degree of abdominal and inner intercostal contraction during the exhalation, or both. The exercises include intercostal stretching and thoracic spine mobility, which aid in abdominothoracic expansion during the inhalation. Other corrective exercises enforced maximal abdominal contraction during the exhalation, teaching active expiration and enhancing the ROM.

This change also resulted in a longer breath hold time. BH improved from pre- to post-intervention, from 37.2 (18.7) to 66.0 (26.9), with a mean change of 24.3 (15.3) (p < 0.001). Although not initially a focus of this study, it was quite interesting to find that breath hold almost doubled post-intervention. This result is in accordance with prior research by Dr. Rosalba Courtney, which demonstrated that lower breath hold time was correlated with apical breathers (Courtney and Cohen, 2008; Courtney et al., 2011c).

### Breathing IQ as an anthropometric index of abdominothoracic flexibility and diaphragmatic breathing efficiency

Recent studies have found an alarmingly high rate of biomechanical breathing dysfunction related to the overuse of upper-thoracic auxiliary muscles in place of the diaphragm (Zikora, Shimozama). This dysfunction causes subsequent fallout ripples throughout the whole body from top to bottom, contributing to or exacerbating chronic back and neck pain (Clifton-Smith, 2014; Hodges et al., 2001), high blood pressure (Radaelli et al., 2023), acid reflux (Eherer et al., 2012), reduced HRV (Courtney et al., 2011a), and panic attacks (Fried, 1999).

The Breathing IQ fills a critical need for an accessible, non-invasive tool to assess biomechanical breathing efficiency. Unlike specialized techniques like ultrasound or the sniff test, the BIQ requires only a flexible tape measure. By empowering a range of preventative health providers with this standardized screening tool and providing immediately actionable correctives, information provided by the BIQ can help address issues at their root cause, potentially reducing unnecessary referrals to specialists, costs, and time and empowering the patient in their own care.

### Addressing concerns in the prior thoracic excursion literature

Current breathing screenings often rely on the subjective observation of breathing rate and fail to capture the full complexity of respiratory biomechanics (Spiteri et al., 1988; Perkins et al., 2005). Although the rate of breath is a common metric in clinical and emergency settings, it has significant potential for errors (Drummond et al., 2020) and fails to consider the mechanical impacts of breathing, despite their direct impacts on the biochemistry and psychology (Courtney, 2017).

The BIQ addresses these limitations by evaluating diaphragmatic efficiency through a combination of thoracic excursion and assessment of dominant breathing musculature to assign a grade from A to F. Rather than relying on a variable “normal” range that does not consider height or weight, the BIQ utilizes a percentage-based approach to calculate the difference between the subject’s inhalation and exhalation, allowing for a personal baseline and approach to progress. By grading individuals based on the LOM and ROM of their breath, the BIQ provides a standardized approach to identify suboptimal biomechanical breathing patterns and inefficient diaphragmatic function. A higher BIQ grade (A) indicates optimal breathing biomechanics and efficient diaphragmatic breathing, while a lower grade (F) suggests impaired breathing mechanics and inefficient diaphragmatic function.

This is a significant improvement over the more traditional focus on simple thoracic excursion as it excludes the impact of reliance on accessory breathing muscles, like the trapezius, SCM, serratus anterior, pectoralis minor, and scalenes, which can reflect hindered diaphragmatic breathing (Wade, 1954).

The BIQ responds to recommendations made by prior studies by standardizing the measurement location and position. For this preliminary study, all participants are assessed while standing, with the tape measure wrapped around the body at the intersection of the vertical mammary papilla line and the inferior rib cage margin. The mean of three maximal inhalation and exhalation measurements is recorded, conducted directly on the skin or over form-fitting clothing. The BIQ is a valuable tool for a wide range of healthcare professionals, including primary care practitioners, physiotherapists, respiratory therapists, and fitness trainers.

## Conclusion

The rate of BPD rooted in faulty breathing mechanics may be significantly more prevalent than previously considered. Our modern lifestyle has led to a decline in diaphragmatic breathing, often replacing it with less efficient apical breathing. This has far-reaching consequences for the body, affecting everything from musculoskeletal function to emotional wellbeing. The diaphragm, a crucial muscle for balance, digestion, movement, spinal health, and even emotional regulation, is often underutilized due to stress-induced muscular bracing and misconceptions about deep breathing. A more common apical breathing pattern can contribute to various health issues, including knee and ankle injuries, digestive problems, and back pain.

Current respiratory evaluations typically involve costly, invasive, operator-dependent assessments. The assessment of BPD, which traditionally has focused on hyperventilation and rate of breathing, could greatly benefit from the inclusion of a brief musculoskeletal screening such as the BIQ. This paper introduces the BIQ, an accessible, noninvasive, affordable tool with corrective exercises that provides patients and clinicians with immediate feedback on breathing mechanics and diaphragmatic breathing efficiency. Joining the location of breathing movement (LOM) with existing thoracic excursion metrics (ROM) provides a significantly more comprehensive understanding of a mechanically unsound breath as the expansion of the chest without the consideration of the muscles powering the movement is deceptive. The BIQ considers optimal thoracic excursion that is achieved using the most efficient primary breathing muscles.

This preliminary study evaluated the effectiveness of the BIQ and its associated corrective exercises in the general population. In a total of N = 384 subjects, before the intervention, 249 (64.8%) were graded an F and only 14 (3.7%) were graded an A. Post-intervention, only 20 (5.2%) were graded an F, and 102 (26.6%) were graded an A. These significant improvements in breathing mechanics in a single 90-minute intervention reflect an improvement in the location of breathing movement from apical to diaphragmatic breathing and a greater degree of abdominothoracic expansion and retraction during inhalation and exhalation.

The proposal and development of this standardized functional breathing tool are timely, given our projection of the state of respiratory health and the burden of respiratory disease on hospital staff. The BIQ can standardize the screening of faulty breathing mechanics and offer a corrective protocol that is intuitive and empowering to the patient. The assessment can be conducted by a range of clinical personnel and in a wide variety of contexts, whether bedside or remotely. Most importantly, reassessment and progress tracking can even be carried out by patients themselves.

## Limitations and future directions

The present study has several limitations. First, diaphragm movement was not assessed using ultrasound, which is the current gold standard for evaluating diaphragm excursion (Marques et al., 2024). A current study by these same authors is underway, comparing BIQ grades and ultrasound measured diaphragm excursion. Second, the results in this study are from subjects without breathing problems, so the results could be indicative but cannot be generalized to individuals with diagnosed breathing dysfunctions. Future research will assess patients with clinical respiratory dysfunction. Third, the fatigue effect of breathing could be an influencing factor in the results as there was no randomization of the order of LOM, ROM, and BH assessments. Future experiments will randomize this order. Fourth, inter-rater reliability was not assessed; this will be done in the subsequent study. Fifth, the authors recognize the limitations to a retrospective study. Subsequent studies will be designed as randomized controlled trials and expand evaluations to include the SEBQ, incentive spirometry, end tidal CO_2_, and HRV to provide greater insights into the relationships between breathing mechanics and physical and mental health.

## Data Availability

The raw data supporting the conclusions of this article will be made available by the authors, without undue reservation.
